# Thin Air, Thick Vessels: Historical and Current Perspectives on Hypoxic Pulmonary Hypertension

**DOI:** 10.3389/fmed.2019.00093

**Published:** 2019-05-01

**Authors:** Jason M. Young, David R. Williams, A. A. Roger Thompson

**Affiliations:** ^1^Edinburgh Medical School, University of Edinburgh, Edinburgh, United Kingdom; ^2^Apex (Altitude Physiology Expeditions), Edinburgh, United Kingdom; ^3^Department of Infection, Immunity and Cardiovascular Disease, University of Sheffield, Sheffield, United Kingdom

**Keywords:** hypoxia, pulmonary hypertension, altitude, vascular remodeling, hypoxic pulmonary vasoconstriction (HPV)

## Abstract

The association between pulmonary hypertension (PH) and hypoxia is well-established, with two key mechanistic processes, hypoxic pulmonary vasoconstriction and hypoxia-induced vascular remodeling, driving changes in pulmonary arterial pressure. In contrast to other forms of pulmonary hypertension, the vascular changes induced by hypoxia are reversible, both in humans returning to sea-level from high altitude and in animal models. This raises the intriguing possibility that the molecular drivers of these hypoxic processes could be targeted to modify pulmonary vascular remodeling in other contexts. In this review, we outline the history of research into PH and hypoxia, before discussing recent advances in our understanding of this relationship at the molecular level, focussing on the role of the oxygen-sensing transcription factors, hypoxia inducible factors (HIFs). Emerging links between HIF and vascular remodeling highlight the potential utility in inhibiting this pathway in pulmonary hypertension and raise possible risks of activating this pathway using HIF-stabilizing medications.

## Introduction

Pulmonary hypertension (PH) is a feature of several distinct clinical phenotypes which, by differing means, result in increased pressure within the pulmonary vasculature. Despite some advancements in treatment over recent years (1), most forms of PH are progressive and life-limiting. In the current classification of PH etiology, Group III (PH due to lung diseases and/or hypoxia) is the second commonest cause of elevated pulmonary artery pressure, behind heart disease (2). Group III encompasses a broad range of conditions such as chronic obstructive pulmonary disease (COPD), interstitial lung disease (ILD) and sleep apnoea (3). Alongside parenchymal changes, two key pathological process, pulmonary vascular remodeling and vasoconstriction, contribute to PH in this group of patients but treatment with pulmonary vasodilators has, to date, been disappointing. New approaches to the management of these patients are thus urgently required to improve outcomes as 3 year survival remains as low as 33% for COPD patients with mean pulmonary artery pressures >40 mmHg (1, 2, 4). While pathologic mechanisms might vary depending on the underlying disease or phenotype, a better understanding of the defining component of Group III disorders, hypoxia, may help provide new targets for therapies.

A causal relationship between hypoxia and PH is well established; hypoxia is frequently used to both precipitate PH in animal models (5) and to induce aberrant cell phenotypes *in vitro* (6). These approaches have greatly improved our understanding of the underlying physiological mechanisms that drive the pathology. In humans, compelling evidence of the effects of hypoxia on pulmonary vascular tone and remodeling derives from studies performed at altitude, where the inherent reduction in barometric pressure results in hypobaric hypoxia. This approach is advantageous for evaluation of the effects of hypoxia on the pulmonary vasculature in relative isolation, without the complicating factors of disease. In this review, we outline the historical context of research into PH and hypoxia and discuss emerging molecular mechanisms for this relationship. We focus on the role of the oxygen-sensing transcription factors, hypoxia inducible factors (HIFs), and links between HIFs and vascular remodeling.

## Important Definitions

Before embarking on this review, it is important to consider the definitions of PH used within this manuscript and others. The term PH is used to describe elevation in mean pulmonary artery pressure (mPAP) from any cause. PH was first classified as a mPAP exceeding 25 mmHg at the 1st World Symposium on Pulmonary Hypertension (WSPH) in 1973 (7). Notably, at the recent 6th WSPH, the upper limit of normal for mPAP was set at 20 mmHg, argued in part due to emerging evidence of poorer survival in patients with mPAPs of 21–24 mmHg and in part based on the distribution of values in healthy population data (8). For a diagnosis of pre-capillary pulmonary hypertension, of any cause, an increased pulmonary vascular resistance (PVR > 3 WU) is also required (8). Pre-capillary hemodynamics that meet the above definition, are not uncommon in patients with lung disease (4, 9), but the prevalence of increased PVR in healthy individuals who are hypoxic without lung disease, for example altitude residents and those with sleep apnoea, is less clear and will be discussed later (10). To avoid confusion we have, where possible, included values (±SD) from the cited literature indicating recorded pulmonary artery pressures and/or PVR.

## Pulmonary Hypertension: A History

Pathological changes in the pulmonary arteries co-existing with right ventricular hypertrophy (RVH) were first observed by the German physician Ernst von Romberg toward the end of the nineteenth century, which he coined “pulmonary vascular sclerosis” (11). However, the etiology of PH remained elusive at this time and was wrongly attributed to syphilis for many years (12, 13). Whilst the British cardiologist Oscar Brenner eventually disproved this link in 1935, he could not provide an explanation for pulmonary vascular changes coinciding with RVH (14). It was only with the advent of right heart catheterization in the mid-twentieth century that these observations were intrinsically linked by raised pulmonary artery pressure (PAP). Despite extensive use in animals in the early twentieth century, cardiac catheterization in humans was widely considered unsafe until Werner Forssman's gallant self-catheterization of his right heart in 1929 (15, 16). Whilst this act of bravery was initially poorly received and widely ignored by the medical community, American physicians Dickinson Richards and Andrew Cournard would recognize the importance of Forssman's work in the 1940s. Their pioneering research characterized mPAP in cardiac and pulmonary diseases for the first time, a feat for which they were awarded a Nobel Prize, together with Forssman, in 1956 (17, 18).

Further work in the 1950s began to establish the clinical and pathological features of PH. In 1951, one of the first detailed descriptions of the haemodynamic profiles of the disease was provided by David Dresdale who also observed cyanosis, orthopnoea and haemoptysis amongst patients with idiopathic PH. Dresdale and others termed their findings “primary pulmonary hypertension” (19, 20); this terminology provided important nomenclature for the emerging research community. Additionally, an extensive characterization of histological changes in PH was described by Donald Heath who, in collaboration with William Whitaker, first detailed extensive thickening of the pulmonary arterial wall associated with fibrosis in 1953, amongst individuals with congenital heart disease, mitral stenosis and idiopathic PH (21, 22). Heath and Jesse Edwards subsequently produced a detailed histological classification system correlated to PH severity in Eisenmenger's syndrome, which ranged from early vascular medial hypertrophy in mild PH to late intimal fibrosis in severe disease (23).

## Early Links Between Acute Hypoxia and Pulmonary Hypertension

Despite elevated PAP being first associated with ventilatory failure in 1852 (24), a causal relationship between hypoxia and PH only became established in 1946 when von Euler and colleagues demonstrated increased mPAP on exposing cats to both hypoxia and hypercapnia (25); in 1947, Dresdale reported similar findings in humans (26). These reports constituted the first measurements of pulmonary arteriole constriction to hypoxia, or hypoxic pulmonary vasoconstriction (HPV), a phenotype which contrasts the vasodilating properties of hypoxia on the systemic circulation (27). At the time, von Euler correctly hypothesized that this physiological response is beneficial in order to shunt blood from areas of regional lung hypoxia that stems from reduced ventilation, thus maintaining blood oxygenation (a concept now termed ventilation-perfusion matching).

However, the adverse effects of this response in the context of more global alveolar hypoxia soon became apparent, particularly in relation to high-altitude pulmonary oedema (HAPE). Whilst a syndrome of cough, blood-stained sputum and severe breathlessness was previously recognized in high altitude sojourners, Hurtado was the first to attribute this to pulmonary oedema in 1937 (28). PH was first identified as co-existing with HAPE in 1962 by Fred et al. (29) in one patient with a mPAP of 46 mmHg, although Hultgren and Spickard had proposed this association in 1960, providing clinical descriptions of a loud second heart sound and electrographic changes consistent with PH in 41 cases of HAPE in Peru (30). Hultgren et al. subsequently confirmed this in seven individuals following acute exposure to high altitude in 1964, in whom mPAP ranged from 33 to 117 mmHg (PVR reported in 2 patients; 8 and 36 WU). Importantly, the authors could also demonstrate a degree of reversibility of pulmonary oedema and elevated mPAP on administration of 100% oxygen (31). Further work from this group, along with others (32), identified a predisposition to pulmonary oedema amongst five individuals with mPAPs of 38.8 ± 10.3 mmHg on ascent to 3,100 m (33).

Despite the early identification of PH as a factor in the pathogenesis of HAPE, how this results in oedema formation remains unclear. Hultgren proposed that because HPV is heterogeneous, areas of the lung are over-perfused leading to pulmonary capillary stress failure in HAPE (34). Indeed, subsequent studies in HAPE-susceptible individuals have provided evidence of exaggerated heterogeneity of perfusion (35), whilst haemodynamic studies have also demonstrated elevated pulmonary capillary pressures (19 ± 1 mmHg vs. 13 ± 1 mmHg in controls) and arterial pressures (mPAP 37 ± 2 mmHg vs. 26 ± 1 mmHg in controls) amongst such individuals at high altitude (36). Other factors in HAPE pathogenesis include impaired nitric oxide (NO) biosynthesis and reduced alveolar fluid reabsorption, as reviewed here (37, 38).

## Chronic Hypoxia and Remodeling of the Pulmonary Vasculature

Concurrently, research began to investigate the effects of chronic hypoxia on the pulmonary vasculature of high-altitude populations. This initially began in cattle which often developed significant oedema around the lower chest at high altitude, dubbed “brisket disease,” a condition that caused significant mortality upon ascent. In the 1940s, Rue Jensen first identified right ventricular dilatation and failure co-existing with brisket disease amongst the high-altitude cattle populations in Colorado (39), with further work with Grover, Reeves and Will identifying a positive correlation between the severity of RVH and the degree of raised PAP (40). Further breeding experiments led by Grover and Reeves suggested an autosomal dominant inheritance of HAPH among these cattle (41, 42). In contrast to Hultgren's later findings amongst patients with HAPE (31), 100% oxygen did not fully reverse PH in cattle (40), indicating a lesser role of HPV in PH pathogenesis in the setting of chronic hypoxia. Interestingly, similar findings were documented by Anand et al. amongst a human population, detailing evidence of peripheral oedema and shortness of breath amongst Indian soldiers who had sojourned at altitudes above 5,800 m for 18 weeks. While no measurements were made at altitude, shortly after return to sea level right heart catheter studies on these patients provided evidence of mild pre-capillary pulmonary hypertension, with mPAP and PVR measured as 26.1 ± 4.5 mmHg and 3.41 ± 2.46 WU, respectively (43).

Elevated PAP in human populations at high altitude was first reported in 1956 by Canepa in one of the first reports of human right heart catheterization in Peruvians from Morochoca (4,540 m), recorded as 25 (range 18–29) mmHg amongst 7 highlanders and 34 and 35 mmHg amongst two chronic mountain sickness patients; however, these findings were initially attributed to polycythaemia, abnormal ventilation and increased cardiac output (44). It would take the work of fellow Peruvians Dante Peñaloza and Javier Arias-Stella in the 1960s to demonstrate that PH amongst high altitude populations was associated with remodeling of the pulmonary vasculature (45–47). Earlier work from Peñaloza confirmed elevated mPAP (23 ± 5.1 mmHg) associated with RVH in Peruvians at high altitude (48, 49) and interestingly, also identified PH amongst new born children both at sea level and altitude, with a swift resolution at sea level that was not recognized amongst Peruvian infants (50). Importantly, the authors found no difference in PAWP and CO between residents at sea level and altitude, with PVR elevated at 4.15 ± 2.66 WU in high altitude dwellers (46, 49). Oxygen administration to Peruvian adults resulted in minor reductions in mPAP of 15–20% (45, 47), mirroring prior results in brisket disease (40) and indicating that pulmonary vascular remodeling was primarily responsible for PH in chronic hypoxia. Further weight to this hypothesis was added by Jensen and Alexander, who later demonstrated a linear relationship between medial hypertrophy of the pulmonary arteries and PAP amongst cattle (51). Notably, despite a failure of immediate resolution with oxygen, Peñaloza identified a normalization of mPAP (12 ± 1.9 mmHg) and PVR (1.81 ± 0.44 WU) amongst high altitude populations following 2 years spent at sea level, demonstrating that changes as a result of chronic hypoxic exposure are not permanent (52). Complementing this finding, the Indian soldiers studied by Anand et al., who had developed signs of right heart failure during their altitude sojourn, made a full recovery, with reversal of cardiomegaly and normalization of mPAP (16.3 ± 2.9 mmHg) and PVR pulmonary vascular resistance (1.34 ± 0.48 WU) 12–16 weeks after descent from high altitude (43).

While the above studies in healthy individuals imply that elevated pulmonary artery pressures are found ubiquitously at altitude, whether the magnitude of elevation in healthy altitude residents reaches that which would define pre-capillary PH remains unclear. A recent meta-analysis by Soria et al. revealed an average systolic PAP of 25.3 mmHg across high altitude populations with a wider distribution than amongst lowlanders implying a low prevalence of PH even by the new WHO criteria (8, 10). Furthermore, PVR is seldom reported and a notable limitation of reported PVRs among historical catheterization studies at altitude, is the lack of correction for hematocrit. Resistance to blood flow is dependent upon viscosity as well as vessel dimensions [reviewed by Vanderpool and Naeije (53)], with equations describing the relationship derived from isolated perfused lung experiments involving alterations in haematocrit (54). Thus, reporting of haematocrit is important in determining true PVR (53, 55) and may lead to false assumptions regarding the extent of vascular remodeling in healthy individuals following hypoxic exposure.

Nonetheless, similar to observations in patients with lung disease, there is a sub-population of altitude residents who develop more severe PH. A consensus definition for high altitude PH (HAPH) was reported in 2005, to encompass those at altitude with exaggerated elevation in PAP and signs of RVH and right heart failure (56). HAPH was defined as a mean PAP of >30 mmHg (or systolic PAP > 50 mmHg) in the absence of excessive erythrocytosis (hemoglobin concentration > 19 g/dl for women, > 21 g/dl for men). This definition allowed discrimination between HAPH and chronic mountain sickness (CMS), in which there is excessive erythrocytosis (57, 58). Despite aforementioned epidemiological studies indicating the rarity of HAPH by the above definition amongst high altitude dwellers (10), the study of such individuals may provide important insights into molecular pathways that drive vasoconstrictive and remodeling processes in both hypoxic PH and, potentially, other forms of PAH. However, it could be argued that a revision of the current definition of HAPH, to include haematocrit-corrected PVR, would facilitate this research.

## Inter-Species Variation at High Altitude

Following these results in both humans and cattle, Donald Heath became interested in inter-species variability in the pulmonary vasculature of high-altitude populations. Heath traveled to Cerro de Pasco, Peru (4,330 m) alongside Peter Harris in 1965, in what became the first of many high-altitude research expeditions dedicated to PH research. A descriptive overview of this work is provided by one of this article's authors in Box 1. In 1974, Heath published their research in llamas (*Lama glama*) demonstrating a lack of pulmonary arteriole muscularisation or RVH at altitude, contrasting previous findings in humans and cattle (*Bos taurus*) (59). A similarly thin walled pulmonary vasculature was also identified in the Himalayan yak (*Bos grunniens*) (60), indicating a role of natural selection in the loss of the thick-walled, reactive pulmonary arteries typically characteristic of the Bos genus.

Box 1 **Adaptation to chronic hypoxia in the andes.**

*The recognition of the different biological classes of man and mammals at high altitude is best illustrated by taking a mental stroll around the streets and surrounding countryside of any small town in the high Andes. The studies undertaken demonstrated that there was no single stereotypical man or mammal at high altitude*.

*Cerro de Pasco is a mining community with a population of 70,000 people, situated at an attitude of 4330 m in the central Andes of Peru. In the streets will be a number of lowlanders who may have arrived at high altitude in a matter of hours from Lima on the coast. Approximately 50% will suffer from benign acute mountain sickness mainly characterized by headache, insomnia, anorexia, nausea and dizziness. These symptoms are the consequence of hypobaric hypoxia and may be regarded as the physiological components of early acclimatization*.

*In contrast, most people are native Quechua Indians born and bred in the high Andes. These descendants of the Inca people have very characteristic physical features of skin color with deeply polycythaemic and suffused conjunctiva and lips. Many will have a capacious chest which looks prominent and out of proportion to their short and stocky physique. These native highlanders lead normal busy lives at high altitude. They participate in vigorous games of football at altitudes exceeding the summit of the Matterhorn in the Swiss Alps*.

*Living on the pastures surrounding Cerro de Pasco are examples of indigenous mountain animals such as the llama, alpaca, vicuna and guanaco. These animals have been living on the Andean altiplano for many thousands of years. One cannot help but be impressed by the vigor and activity of these animals in an atmosphere characterized by severe hypobaric hypoxia*.

An interesting biological issue arises when species from within the same genus interbreed; one such example is the interbreeding of cattle giving rise to species such as the dzo (cow x yak) and stol (dzo x bull) (61). In 1986, work from Peter Harris' group identified that protection from PH correlated with the degree of yak heritage; whilst dzos and yaks demonstrated minimal PH, half of the stols had significantly raised PAPs similar to that of cattle (62), indicating a degree of inheritance. These observations lend support to the concept that animals indigenous to high altitude have become genetically adapted to their hypoxic environment, vs. acclimatization as seen in other species.

## Molecular Mechanisms of PH in Acute and Chronic Hypoxic Exposure

While the evidence above clearly illustrates connections between hypoxic exposure and pulmonary hypertension, the underlying genetic, molecular and cellular mechanisms that regulate these phenotypes remain unclear and in part, controversial. Nonetheless, basic science work over the last 25 years has advanced our understanding of common pathways that govern both adaptation to altitude and hypoxia-induced PH. Reviewed extensively elsewhere (63–65), pulmonary vasoconstriction in acute hypoxia comprises at least two phases involving distinct mechanisms. Initially, changes in redox status within smooth muscle cell mitochondria mediate alterations in potassium and voltage-gated calcium channel flux, promoting contraction (63). Subsequently, vasoconstriction is maintained by mechanisms that include reduced bioavailability of NO (66), release of endothelial-derived vasoconstrictors (67) and increases in myofilament calcium sensitivity (68). The focus of this article will be on the role of the hypoxia-inducible factors (HIFs) in vascular remodeling due to chronic hypoxic exposure.

## Hypoxia-Inducible Factors (HIFs) and Pulmonary Vascular Remodeling

**Figure 1:**
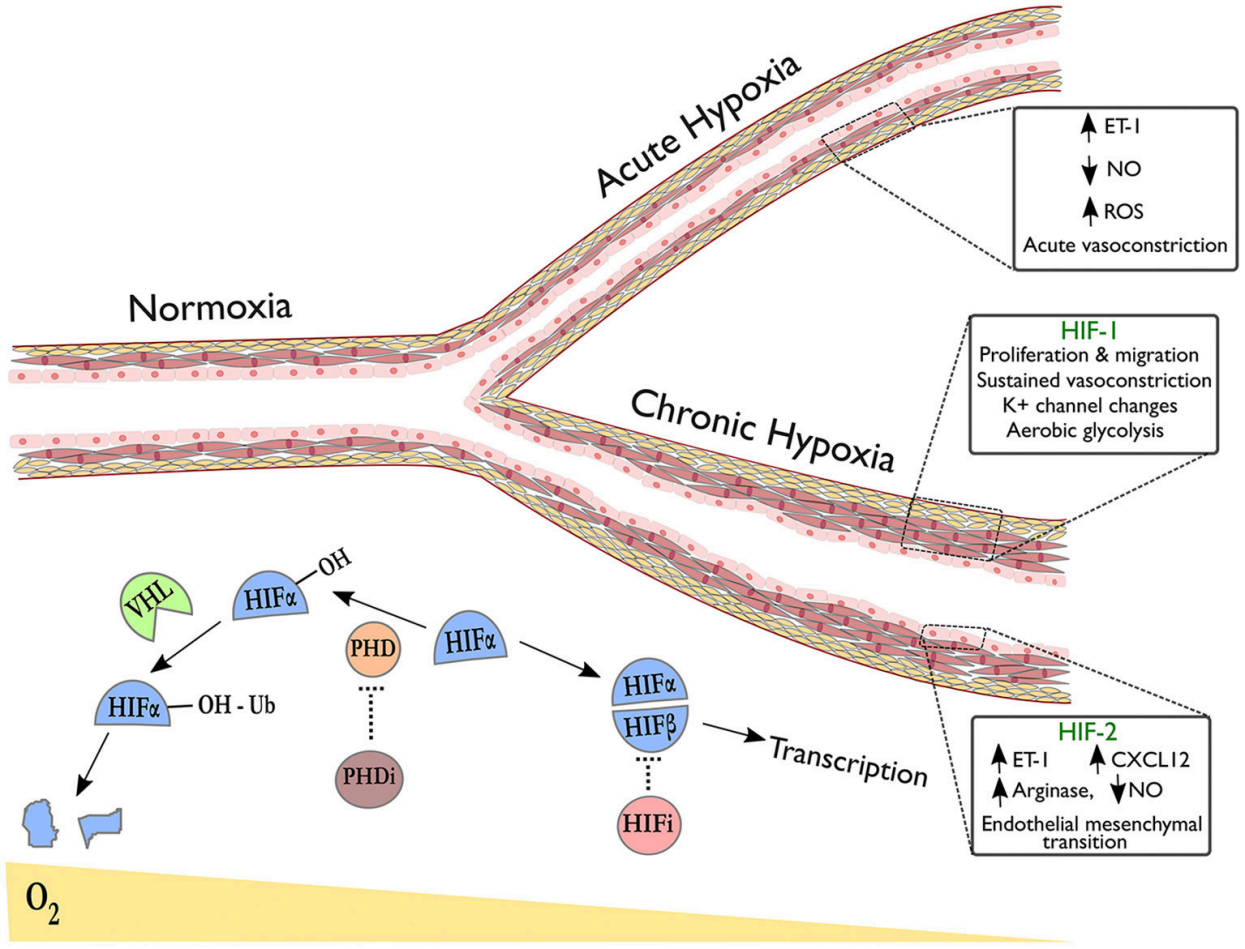
Pulmonary vascular responses to hypoxia with emphasis on the role of HIF isoforms in remodeling. The right upper branch of this vessel depicts vasoconstriction in acute hypoxia, occurring due to alterations in redox and NO signaling and release of vasoactive mediators. The lower branch indicates remodeling in the context of sustained hypoxic exposure and illustrates HIF-dependent processes revealed by tissue-specific deletion of HIF-isoforms in endothelial cells (HIF-2α) or smooth muscle cells (HIF-1α). Below the vessel, a schematic shows degradation of hydroxylated HIF-α subunits in normoxia via the von-Hippel Lindau (VHL) pathway. In hypoxia or following treatment with prolyl hydroxylase inhibitors (PHDi), HIF-α stabilization and dimerization with HIF-β occurs, leading to transcription of target genes. HIF inhibitors (HIFi) with specific activity against HIF-2α are in clinical development.

HIFs are a family of heterodimeric transcription factors, discovered in 1995 (69), whose alpha subunits are stabilized in hypoxia by the inhibition of oxygen-dependent prolyl hydroxylase (PHD) enzyme activity (70); under normoxic conditions, hydroxylation of HIF-alpha by PHDs targets them for ubiquitination by the VHL complex, resulting in subsequent proteasomal degradation (Figure 1) (71). Whilst HIF-1α is expressed ubiquitously throughout body tissues (72), HIF-2α expression is tissue specific with an endothelial bias (73, 74). In hypoxia, stabilization of HIF-α subunits induces transcription of targets with a wide range of functions.

Perhaps unsurprisingly, genetic variation in the HIF pathway has been identified amongst indigenous altitude dwellers. Notably, genome wide association studies in the Tibetan population have identified single nucleotide polymorphisms (SNPs) in *EPAS1* (encoding HIF-2α) and *ELGN1* (encoding PHD2) that were not enriched in lowlanders (75–77). *EPAS1* variants were associated with lower PAP (78) and a high frequency *ELGN1* mutation has been linked to reduced proliferation of erythroid progenitors in response to EPO, thus dampening hypoxia-induced erythrocytosis (79). These findings demonstrate a selection pressure for specific HIF pathway polymorphisms over 25,000 years at altitude that has aided adaptation for the Tibetan population. Interestingly, such variation is not observed in Andean counterparts, a population that has resided at altitude for 15,000 years and who are more susceptible to PH (80, 81) and erythrocytosis (82).

However, the evidence for HIF pathway polymorphisms influencing remodeling processes is weakened by the observation that correction for erythrocytosis reduces mPAP amongst Andean populations to values near those of Tibetans (57). Thus, correcting for erythrocytosis argues against a susceptibility of Andean populations to HIF-mediated remodeling processes. In light of the new PH definition (8), however, Andean corrected mPAP remains consistently above 20 mmHg at rest and the slope of rise in mPAP with cardiac output is steeper than that of lowlanders (57).

Furthermore, evidence from murine models strongly implicates the HIF pathway in hypoxia-induced vascular remodeling. Soon after the discovery of the pathway, early work in both *Hif1a* and *Hif2a* heterozygotes revealed a marked reduction in PH and vascular remodeling following chronic exposure to 10% oxygen (83, 84). Conversely, *HIF2A* (*EPAS1*) gain-of-function mutations can predispose to PH; a *Hif2a* variant in high altitude cattle increases susceptibility to brisket disease (85), whilst a *HIF2A* mutation causing familial erythrocytosis is also associated with elevated systolic PAP in humans (86). A mouse generated to have the same G536W gain-of-function mutation in the *Hif2a* gene also developed erythrocytosis and PH, providing further evidence of cross-species conservation of this HIF-2α role (87). Additionally, both animal models and patients with Chuvash polycythaemia (CP), characterized by a VHL mutation, exhibit marked erythrocytosis and elevated PAP that could be rescued in mice by *Hif2a* but not *Hif1a* deletion (88–90). While the descriptions of elevated PAP in humans with CP did not include right heart catheter data or haematocrit-corrected PVR, it is worth noting that elevations in systolic PAP and vessel muscularisation in young mice with homozygous VHL mutations, preceded the onset of polycythaemia (90).

## Tissue-Specific Manipulation of HIF Expression Reveals Distinct Roles for HIF Isoforms in Pulmonary Vascular Remodeling

Evidence is now emerging as to how the HIF isoforms regulate pulmonary vascular cell function, with advances gained through use of murine tissue-specific deletion models, see Figure 1. For example, HIF-1α has been implicated in both vasoconstriction and vascular cell proliferation, the two key components of hypoxic pulmonary hypertension. Ball et al. demonstrated that inducible *Hif1a* deletion in PASMCs reduced right ventricular systolic pressure, arterial wall thickness and vessel muscularisation in chronic hypoxia (91), whilst Shiekh et al. reported a dependence on *Hif1a* in PASMC progenitors in order to drive distal migration and expansion (92). Proposed mechanisms that could explain these findings include enhanced intracellular calcium via *Hif1a* dependent downregulation of K^+^ channels (93) and upregulation of transient receptor potential calcium channels (94), recognized to enhance vasoconstriction, PASMC proliferation and migration (95). HIF-1α also mediates pro-proliferative metabolic changes in PASMCs and fibroblasts that could contribute to hypoxia-induced remodeling. One widely recognized consequence of HIF signaling amongst cancer cells is the favoring of glycolysis over oxidative phosphorylation in aerobic conditions, known as the “Warburg effect,” inducing glycolytic enzymes to enhance ATP production and promote tumor growth (96, 97). Interestingly, HIF signaling amongst pulmonary arterial smooth muscle cells (PASMCs) and fibroblasts results in a similar shift to aerobic glycolysis as seen in tumors (98–100), with increased glucose uptake observed in the lungs of rats with hypoxia-induced PH and in PAH patients (101, 102). This metabolic reprogramming of pulmonary vascular cells has proven stable *ex vivo* with evidence of underlying epigenetic regulation (103, 104). Targeting these mechanisms may limit hypoxia-induced PASMC proliferation in the pulmonary vasculature.

Consistent with its predominantly endothelial expression profile, a growing body of evidence implicates endothelial cell (EC) HIF-2α expression as essential for pulmonary vascular remodeling through varied biological mechanisms, see Figure 1. Two studies have demonstrated severe and spontaneous PH following *Phd2* knockdown in murine ECs (105, 106). Double knockouts of *Phd2* and either HIF isoform revealed that this was a *Hif2a*-mediated phenotype (105, 106) but the studies highlighted different mechanisms: one associating HIF-2α expression with reduced expression of the potent vasoconstrictor endothelin-1 (ET-1) (106) and the other demonstrating HIF-2α involvement in CXCL12-mediated PASMC proliferation (105). Reduced EC *Phd2* expression was also observed amongst occlusive vessels in IPAH (105), implying relevance to human pathology. Notably, these *Phd2* knockout mice did not develop polycythaemia prior to the development of PH.

The NO synthesis pathway has also been implicated in EC HIF-2α-mediated remodeling. Cowburn et al. observed a similar level of protection from hypoxia-induced PH as *Hif2a* knockdown following EC-specific deletion of arginase-1 (*Arg1*), a downstream HIF-2α target and negative regulator of NO synthesis (107). Additionally, ECs from PH patients demonstrated impaired NO production *in vitro*, restored on arginase inhibition (107). A further mechanism by which HIF-2α could contribute to remodeling is through regulation of endothelial-mesenchymal transition (EMT), a process implicated in pathogenic remodeling (108). Tang et al. showed that markers of EMT were regulated by HIF-2α in ECs and that while endothelial-specific deletion of *Hif2a* protected mice from hypoxia-induced PH, deletion of *Hif2a* in vascular smooth muscle cells did not (109).

There remain notable controversies in the literature surrounding HIF-mediated regulation of remodeling. Whilst Ball et al. demonstrated a role for PASMC *Hif1a* in chronic hypoxic remodeling using a tamoxifen-inducible conditional deletion (91), Kim et al. reported enhanced pulmonary arterial tone in the absence of arterial muscularisation following constitutive PASMC-specific *Hif1a* deletion (110). Similarly, constitutive EC *Hif1a* deletion was found to confer no protection to PH by three authors (105–107), whilst Shiekh et al. could ameliorate PH following tamoxifen-inducible conditional EC *Hif1a* deletion, which prevented PASMC expansion and distal migration (92). Alongside evidence detailing the importance of embryonic HIF signaling for the developing vasculature (111), these observed differences imply a role of early HIF-1α signaling in pulmonary vessel development.

The crucial role of HIF isoforms in hypoxia-induced PH has identified the inhibition of these molecules as an important strategy for targeting remodeling processes. Whilst efforts to develop HIF pathway inhibitors have previously proven challenging due to poor efficacy, HIF isoform specificity and adverse effects (112, 113), a specific HIF-2α small molecule inhibitor developed to treat renal cancer has demonstrated a favorable safety profile in a recent Phase I trial (114). Encouragingly, the use of another HIF-2α inhibitor, C76, has recently been demonstrated to attenuate remodeling in three murine models of PH, with no notable inhibition of HIF-1α (115).

The pleiotropic nature of HIF signaling has identified several other pathways as possible therapeutic targets. Using congenic linkage analysis, Zhao et al. discovered a dependence on intracellular zinc in hypoxia-induced remodeling. Homozygous deletion of the zinc transporter ZIP12, a target of both HIF-1α and HIF-2α, was found to attenuate PAP, RVH and vascular remodeling in chronic hypoxia (116). Additionally, induction of ZIP12 was also reported in the pulmonary tissue in Brisket disease and highland human populations (116). How intracellular zinc influences hypoxia-induced remodeling remains unclear; however, targeting intracellular zinc homeostasis may represent a further therapeutic strategy.

## Conclusions

This article has reviewed historical observations connecting hypoxia and pulmonary hypertension and described more recent insights into the molecular mechanisms involved in hypoxia-induced remodeling. Notably, the evidence linking HIF expression to processes involved in vascular remodeling strongly raises the prospect of HIF inhibition, and in particular HIF-2α inhibition, as a strategy in order to ameliorate vascular pathology in the context of chronic hypoxia. The recent success of a HIF-2α inhibitor in several murine models is supportive of such a strategy and may lead to the consideration of clinical trials amongst PH patients in the future. However, the mechanistic links between HIF-pathway activation and PH, notably the development of spontaneous PH following *Phd2* deletion, should also raise a note of caution for use of PHD inhibitors which are currently undergoing Phase II/III clinical trials in renal anemia (117).

Genetic insights gained through study of high-altitude populations suggests that a greater appreciation of factors underlying altitude adaptation may highlight further mechanisms involved in the regulation of vascular remodeling. However, while similarities exist between the pathological features of hypoxia-induced PH and other forms of the disease, the extent of overlap in the pathological mechanisms, even for patients with chronic respiratory disease, remains unclear. Furthermore, the notable lack of correction for haematocrit in previous work reporting PVR at altitude casts some doubt over some of the apparent differences between altitude populations, which may in fact be due to differences in haematocrit. Nonetheless, a reversal of PH on return to sea level provides the tantalizing possibility that exploiting endogenous mechanisms might provide agents that reverse vascular remodeling in hypoxic disease. Therefore, there is still hope that lessons learned from studying hypoxia-induced disease could impact on the search for agents that target pervasive vascular remodeling in other forms of PH.

## Author Contributions

JY wrote the manuscript. DW and AART drafted additional text and edited the manuscript. All authors approved the final version.

### Conflict of Interest Statement

AART has received funds to attend educational events from Actelion. The remaining authors declare that the research was conducted in the absence of any commercial or financial relationships that could be construed as a potential conflict of interest.

## References

[1] Gall H Felix JF Schneck FK Milger K Sommer N Voswinckel R. The giessen pulmonary hypertension registry: survival in pulmonary hypertension subgroups. J Hear Lung Transplant. (2017) 36:957–67. 10.1016/j.healun.2017.02.016 28302503

[2] Strange G Playford D Stewart S Deague JA Nelson H Kent A. Pulmonary hypertension: prevalence and mortality in the Armadale echocardiography cohort. Heart. (2012) 98:1805–11. 10.1136/heartjnl-2012-301992 22760869 PMC3533383

[3] Simonneau G Gatzoulis MA Adatia I Celermajer D Denton C Ghofrani A. Updated clinical classification of pulmonary hypertension. J Am Coll Cardiol. (2013) 62:D34–41. 10.1016/j.jacc.2013.10.029 24355639

[4] Hurdman J Condliffe R Elliot CA Swift A Rajaram S Davies C. Pulmonary hypertension in COPD: results from the ASPIRE registry. Eur Respir J. (2013) 41:1292–301. 10.1183/09031936.00079512 23018917

[5] Maarman G Lecour S Butrous G Thienemann F Sliwa K. A comprehensive review: the evolution of animal models in pulmonary hypertension research; are we there yet? Pulm Circ. (2013) 3:739–56. 10.1086/674770 25006392 PMC4070827

[6] Pugliese SC Poth JM Fini MA Olschewski A El Kasmi KC Stenmark KR. The role of inflammation in hypoxic pulmonary hypertension: from cellular mechanisms to clinical phenotypes. Am J Physiol Lung Cell Mol Physiol. (2015) 308:L229–52. 10.1152/ajplung.00238.2014 25416383 PMC4338929

[7] Hatano S Strasser T World Health Organization Primary Pulmonary Hypertension: Report on a WHO Meeting, Geneva, 15-17 October 1973. Geneva: World Health Organization (1975).

[8] Galiè N McLaughlin V V Rubin LJ Simonneau G. An overview of the 6th World Symposium on Pulmonary Hypertension. Eur Respir J. (2019) 53:1802148. 10.1183/13993003.02148-2018 30552088 PMC6351332

[9] Seeger W Adir Y Barberà JA Champion H Coghlan JG Cottin V. Pulmonary hypertension in chronic lung diseases. J Am Coll Cardiol. (2013) 62:D109–16. 10.1016/j.jacc.2013.10.036 24355635

[10] Soria R Egger M Scherrer U Bender N Rimoldi SF Rimoldi SF. Pulmonary artery pressure and arterial oxygen saturation in people living at high or low altitude: systematic review and meta-analysis. J Appl Physiol. (2016) 121:1151–9. 10.1152/japplphysiol.00394.2016.-More 27660297

[11] Park MH Historical perspective on the classification and nomenclature of pulmonary hypertension. In: Maron B Zamanian R Waxman A editors. Pulmonary Hypertension. Cham: Springer International Publishing (2016). p. 3–15.

[12] Escudero P The Black Cardiacs and the Ayerza's disease. Rev Crit. (1911).

[13] Mazzei JA Mazzei ME. A tribute: Abel Ayerza and pulmonary hypertension. Eur Respir Rev. (2011) 20:220–1. 10.1183/09059180.00006811 22130814 PMC9487740

[14] Brenner O Pathology of the vessels of the pulmonary circulation. Arch Intern Med. (1935) 56:211 10.1001/archinte.1935.03920020003001

[15] Forssmann W Die Sondierung des Rechten Herzens. Klin Wochenschr. (1929) 8:2085–7. 10.1007/BF01875120

[16] van Wolferen SA Grünberg K Vonk Noordegraaf A Diagnosis and management of pulmonary hypertension over the past 100 years. Respir Med. (2007) 101:389–98. 10.1016/J.RMED.2006.11.022 17222544

[17] Cournand A Control of the pulmonary circulation in man with some remarks on methodology. In: Nobel Lectures: Physiology or Medicine. Amsterdam: Elsevier Publishing (1964). p. 529–42.

[18] Richards DW. The contributions of right heart catheterization to physiology and medicine, with some observations on the physiopathology of pulmonary heart disease. Am Heart J. (1957) 54:161–71. 10.1016/0002-8703(57)90143-6 13444181

[19] Dresdale DT Schultz M Michtom RJ. Primary pulmonary hypertension. Am J Med. (1951) 11:686–705. 10.1016/0002-9343(51)90020-4 14902839

[20] Gilmour JR Evans W. Primary pulmonary hypertension. J Pathol Bacteriol. (1946) 58:687–97. 10.1002/path.1700580410 20297309

[21] Heath D Whitaker W. Hypertensive pulmonary vascular disease. Circulation. (1956) 14:323–43. 10.1161/circ.14.3.323 13365044

[22] Whitaker W. The initiation of an interest in the pulmonary circulation. Thorax. (1994) 49:S2–4. 10.1136/thx.49.Suppl.S2 7974322 PMC1112573

[23] Heath D Edwards J. The pathology of hypertensive pulmonary vascular disease. Circulation. (1958) 18:533–47. 10.1161/circ.18.4.533 13573570

[24] Beutner CA Ueber die Strom-und Druckkrafte des Blutes in der Arteria pulmonalis. Z Ration Med. (1852) 2:97–138.

[25] Euler USV Liljestrand G Observations on the pulmonary arterial blood pressure in the cat. Acta Physiol Scand. (1946) 12:301–20. 10.1111/j.1748-1716.1946.tb00389.x

[26] Motley HL Cournand A Werko L Himmelstein A Dresdale D. The influence of short periods of induced acute anoxia upon pulmonary artery pressures in man. Am J Physiol Content. (1947) 150:315–20. 10.1152/ajplegacy.1947.150.2.315 20258388

[27] Kulandavelu S Balkan W Hare JM. Regulation of oxygen delivery to the body via hypoxic vasodilation. Proc Natl Acad Sci USA. (2015) 112:6254–5. 10.1073/pnas.1506523112 25944936 PMC4443334

[28] Hurtado A Aspectos Fisiologicos y Patologicos de la Vida en la Altura. Rev Med Peru. (1937) 9:3–52.

[29] Fred HL Schmidt AM Bates T Hecht HH Acute pulmonary edema of altitude: clinical and physiologic observations. Circulation. (1962) 25:929–37. 10.1161/01.CIR.25.6.929

[30] West JB High Life : A History of High-Altitude Physiology and Medicine. New York: Springer (1998).

[31] Hultgren HN Lopez CE Lundberg E Miller H. Physiologic studies of pulmonary edema at high altitude. Circulation. (1964) 29:393–408. 10.1161/01.CIR.29.3.393 14131411

[32] Viswanathan R Jain SK Subramanian S Subramanian TAV Dua GL Giri J Pulmonary Edema of High Altitude. Am Rev Respir Dis. (1969) 100:334–41. 10.1164/arrd.1969.100.3.334 5822044

[33] Hultgren HN Grover RF Hartley LH. Abnormal circulatory responses to high altitude in subjects with a previous history of high-altitude pulmonary edema. Circulation. (1971) 44:759–70. 10.1161/01.CIR.44.5.759 5115068

[34] Hultgren HN. High-altitude pulmonary edema: current concepts. Annu Rev Med. (1996) 47:267–84. 10.1146/annurev.med.47.1.267 8712781

[35] Dehnert C Risse F Ley S Kuder TA Buhmann R Puderbach M. Magnetic resonance imaging of uneven pulmonary perfusion in hypoxia in humans. Am J Respir Crit Care Med. (2006) 174:1132–8. 10.1164/rccm.200606-780OC 16946125

[36] Maggiorini M Mélot C Pierre S Pfeiffer F Greve I Sartori C. High-altitude pulmonary edema is initially caused by an increase in capillary pressure. Circulation. (2001) 103:2078–83. 10.1161/01.CIR.103.16.2078 11319198

[37] Scherrer U Rexhaj E Jayet PY Allemann Y Sartori C. New insights in the pathogenesis of high-altitude pulmonary edema. Prog Cardiovasc Dis. (2010) 52:485–92. 10.1016/j.pcad.2010.02.004 20417341

[38] Swenson ER Bärtsch P. High-altitude pulmonary edema. Comprehen Physiol. (2012) 2: 2753–73. 10.1002/cphy.c100029 23720264

[39] Rhodes J. Comparative physiology of hypoxic pulmonary hypertension: historical clues from brisket disease. J Appl Physiol. (2005) 98:1092–100. 10.1152/japplphysiol.01017.2004 15703167

[40] Alexander AF Will DH Grover RF Reeves JT. Pulmonary hypertention and right ventricular hypertrophy in catle at high altitude. Am J Vet Res. (1960) 21:199–204. 13792577

[41] Will DH Hicks JL Card CS Alexander AF. Inherited susceptibility of cattle to high-altitude pulmonary hypertension. J Appl Physiol. (1975) 38:491–4. 10.1152/jappl.1975.38.3.491 238929

[42] Weir EK Tucker A Reeves JT Will DH Grover RF. The genetic factor influencing pulmonary hypertension in cattle at high altitude. Cardiovasc Res. (1974) 8:745–9. 4457227 10.1093/cvr/8.6.745

[43] Anand IS Chandrashekhar Y Bali HK Wahi PL Jindal SK Malhotra RM Adult subacute mountain sickness—a syndrome of congestive heart failure in man at very high altitude. Lancet. (1990) 335:561–5. 10.1016/0140-6736(90)90348-9 1968575

[44] Rotta A Cánepa A Hurtado A Velásquez T Chávez R. Pulmonary circulation at sea level and at high altitudes. J Appl Physiol. (1956) 9:328–36. 10.1152/jappl.1956.9.3.328 13376451

[45] Arias-Stella J Saldana M. The terminal portion of the pulmonary arterial tree in people native to high altitudes. Circulation. (1963) 28:915–25. 10.1161/01.CIR.28.5.915 14079195

[46] Penaloza D Arias-Stella J. The heart and pulmonary circulation at high altitudes: healthy highlanders and chronic mountain sickness. Circulation. (2007) 115:1132–46. 10.1161/CIRCULATIONAHA.106.624544 17339571

[47] Arias-Stella J Castillo Y. The muscular pulmonary arterial branches in stillborn natives of high altitude. Lab Invest. (1966) 15:1951–9. 5960463

[48] Peñaloza D Gamboa R Marticorena E Echevarría M Dyer J Gutierrez E. The influence of high altitudes on the electrical activity of the heart. Am Heart J. (1961) 61:101–15. 10.1016/0002-8703(61)90522-1 13734073

[49] Peñaloza D Sime F Banchero N Gamboa R Cruz J Marticorena E Pulmonary hypertension in healthy men born and living at high altitudes. Am J Cardiol. (1963) 11:150–7. 10.1016/0002-9149(63)90055-9 13992990

[50] Peñaloza D Gamboa R Dyer J Echevarría M Marticorena E. The influence of high altitudes on the electrical activity of the heart. I. Electrocardiographic and vectocardiographic observations in the newborn, infants, and children. Am Heart J. (1960) 59:111–28. 10.1016/0002-8703(60)90390-2 14431548

[51] Alexander AF Jensen R. Pulmonary vascular pathology of high-altitude induced pulmonary hypertension in cattle. Am J Vet Res. (1963) 24:1112–22. 14081444

[52] Sime F Peñaloza D Ruiz L. Bradycardia, increased cardiac output, and reversal of pulmonary hypertension in altitude natives living at sea level. Br Heart J. (1971) 33:647–57. 10.1136/hrt.33.5.647 5115010 PMC487232

[53] Vanderpool RR Naeije R. Hematocrit-corrected pulmonary vascular resistance. Am J Respir Crit Care Med. (2018) 198:305–9. 10.1164/rccm.201801-0081PP 29537290

[54] Linehan JH Haworth ST Nelin LD Krenz GS Dawson CA. A simple distensible vessel model for interpreting pulmonary vascular pressure-flow curves. J Appl Physiol. (1992) 73:987–94. 10.1152/jappl.1992.73.3.987 1400067

[55] Hoffman JIE. Pulmonary vascular resistance and viscosity: the forgotten factor. Pediatr Cardiol. (2011) 32:557–61. 10.1007/s00246-011-9954-3 21432030

[56] León-Velarde F Maggiorini M Reeves JT Aldashev A Asmus I Bernardi L. Consensus statement on chronic and subacute high altitude diseases. High Alt Med Biol. (2005) 6:147–57. 10.1089/ham.2005.6.147 16060849

[57] Naeije R Vanderpool R. Pulmonary hypertension and chronic mountain sickness. High Alt Med Biol. (2013) 14:117–25. 10.1089/ham.2012.1124 23795731

[58] Villafuerte FC Corante N. Chronic mountain sickness: clinical aspects, etiology, management, and treatment. High Alt Med Biol. (2016) 17:61–9. 10.1089/ham.2016.0031 27218284 PMC4913504

[59] Heath D Smith P Williams D Harris P Arias-Stella J Krüger H. The heart and pulmonary vasculature of the llama (Lama glama). Thorax. (1974) 29:463–71. 10.1136/thx.29.4.463 4854860 PMC470181

[60] Heath D Williams D Dickinson J. The pulmonary arteries of the yak. Cardiovasc Res. (1984) 18:133–139. 10.1093/cvr/18.3.133 6705004

[61] Porter V Alderson L Hall S Sponenberg P Mason's World Encyclopedia of Livestock Breeds and Breeding: 2 Volume Pack. Wallington: CAB International (2016).

[62] Anand IS Harris E Ferrari R Pearce P Harris P. Pulmonary haemodynamics of the yak, cattle, and cross breeds at high altitude. Thorax. (1986) 41:696–700. 10.1136/thx.41.9.696 3787555 PMC460434

[63] Dunham-Snary KJ Wu D Sykes EA Thakrar A Parlow LRG Mewburn JD. Hypoxic pulmonary vasoconstriction: from molecular mechanisms to medicine. Chest. (2017) 151:181–92 10.1016/j.chest.2016.09.001 27645688 PMC5310129

[64] Wilkins MR Ghofrani H-A Weissmann N Aldashev A Zhao L. Pathophysiology and treatment of high-altitude pulmonary vascular disease. Circulation. (2015) 131:582–90. 10.1161/CIRCULATIONAHA.114.006977 25666980

[65] Siques P Brito J Pena E. Reactive oxygen species and pulmonary vasculature during hypobaric hypoxia. Front Physiol. (2018) 9:865. 10.3389/fphys.2018.00865 30050455 PMC6052911

[66] Bailey DM Dehnert C Luks AM Menold E Castell C Schendler G. High-altitude pulmonary hypertension is associated with a free radical-mediated reduction in pulmonary nitric oxide bioavailability. J Physiol. (2010) 588:4837–47. 10.1113/jphysiol.2010.194704 20876202 PMC3010150

[67] Kourembanas S Marsden PA McQuillan LP Faller DV. Hypoxia induces endothelin gene expression and secretion in cultured human endothelium. J Clin Invest. (1991) 88:1054–7. 10.1172/JCI115367 1885767 PMC295521

[68] Weigand L Shimoda LA Sylvester JT. Enhancement of myofilament calcium sensitivity by acute hypoxia in rat distal pulmonary arteries. Am J Physiol Lung Cell Mol Physiol. (2011) 301:L380–7. 10.1152/ajplung.00068.2011 21665962 PMC3174742

[69] Wang GL Jiang BH Rue EA Semenza GL. Hypoxia-inducible factor 1 is a basic-helix-loop-helix-PAS heterodimer regulated by cellular O2 tension. Proc Natl Acad Sci USA. (1995) 92:5510–4. 10.1073/pnas.92.12.5510 7539918 PMC41725

[70] Jaakkola P Mole DR Tian YM Wilson MI Gielbert J Gaskell SJ. Targeting of HIF-alpha to the von Hippel-Lindau ubiquitylation complex by O2-regulated prolyl hydroxylation. Science. (2001) 292:468–72. 10.1126/science.1059796 11292861

[71] Maxwell PH Wiesener MS Chang G-W Clifford SC Vaux EC Cockman ME. The tumour suppressor protein VHL targets hypoxia-inducible factors for oxygen-dependent proteolysis. Nature. (1999) 399:271–5. 10.1038/20459 10353251

[72] Semenza GL. Hypoxia-inducible factor 1: master regulator of O2 homeostasis. Curr Opin Genet Dev. (1998) 8:588–94. 9794818 10.1016/s0959-437x(98)80016-6

[73] Patel SA Simon MC. Biology of hypoxia-inducible factor-2alpha in development and disease. Cell Death Differ. (2008) 15:628–34. 10.1038/cdd.2008.17 18259197 PMC2882207

[74] Tian H McKnight SL Russell DW. Endothelial PAS domain protein 1 (EPAS1), a transcription factor selectively expressed in endothelial cells. Genes Dev. (1997) 11:72–82. 10.1101/gad.11.1.72 9000051

[75] Beall CM Cavalleri GL Deng L Elston RC Gao Y Knight J Natural selection on EPAS1 (HIF2) associated with low hemoglobin concentration in Tibetan highlanders. Proc Natl Acad Sci USA. (2010) 107:11459–64. 10.1073/pnas.1002443107 20534544 PMC2895075

[76] Yi X Liang Y Huerta-Sanchez E Jin X Cuo ZXP Pool JE. Sequencing of 50 human exomes reveals adaptation to high altitude. Science. (2010) 329:75–8. 10.1126/science.1190371 20595611 PMC3711608

[77] Xiang K Ouzhuluobu Peng Y Yang Z Zhang X Cui C. Identification of a Tibetan-specific mutation in the hypoxic gene EGLN1 and its contribution to high-altitude adaptation. Mol Biol Evol. (2013) 30:1889–98. 10.1093/molbev/mst090 23666208

[78] Peng Y Cui C He Y Ouzhuluobu Zhang H Yang D. Down-regulation of EPAS1 transcription and genetic adaptation of tibetans to high-altitude hypoxia. Mol Biol Evol. (2017) 34:818–30. 10.1093/molbev/msw280 28096303 PMC5400376

[79] Lorenzo FR Huff C Myllymäki M Olenchock B Swierczek S Tashi T. A genetic mechanism for Tibetan high-altitude adaptation. Nat Genet. (2014) 46:951–6. 10.1038/ng.3067 25129147 PMC4473257

[80] Groves BM Sutton J Droma T McCullough RG McCullough RE Zhuang J. Minimal hypoxic pulmonary hypertension in normal Tibetans at 3,658 m. J Appl Physiol. (1993) 74:312–8. 10.1152/jappl.1993.74.1.312 8444708

[81] Gupta ML Rao KS Anand IS Banerjee AK Boparai MS. Lack of smooth muscle in the small pulmonary arteries of the native Ladakhi: is the Himalayan highlander adapted? Am Rev Respir Dis. (1992) 145:1201–4. 10.1164/ajrccm/145.5.1201 1586066

[82] Beall CM. Two routes to functional adaptation: Tibetan and Andean high-altitude natives. Proc Natl Acad Sci USA. (2007) 104:8655–60. 10.1073/pnas.0701985104 17494744 PMC1876443

[83] Yu AY Shimoda LA Iyer N V Huso DL Sun X McWilliams R. Impaired physiological responses to chronic hypoxia in mice partially deficient for hypoxia-inducible factor 1alpha. J Clin Invest. (1999) 103:691–6. 10.1172/JCI5912 10074486 PMC408131

[84] Brusselmans K Compernolle V Tjwa M Wiesener MS Maxwell PH Collen D. Heterozygous deficiency of hypoxia-inducible factor−2α protects mice against pulmonary hypertension and right ventricular dysfunction during prolonged hypoxia. J Clin Invest. (2003) 111:1519–27. 10.1172/JCI200315496 12750401 PMC155039

[85] Newman JH Holt TN Cogan JD Womack B Phillips JA Li C. Increased prevalence of EPAS1 variant in cattle with high-altitude pulmonary hypertension. Nat Commun. (2015) 6:6863. 10.1038/ncomms7863 25873470 PMC4399003

[86] Tan Q Kerestes H Percy MJ Pietrofesa R Chen L Khurana TS. Erythrocytosis and pulmonary hypertension in a mouse model of human HIF2A gain of function mutation. J Biol Chem. (2013) 288:17134–44. 10.1074/jbc.M112.444059 23640890 PMC3682519

[87] Gale DP Harten SK Reid CDL Tuddenham EGD Maxwell PH. Autosomal dominant erythrocytosis and pulmonary arterial hypertension associated with an activating HIF2 alpha mutation. Blood. (2008) 112:919–21. 10.1182/blood-2008-04-153718 18650473

[88] Bushuev VI Miasnikova GY Sergueeva AI Polyakova LA Okhotin D Gaskin PR. Endothelin-1, vascular endothelial growth factor and systolic pulmonary artery pressure in patients with Chuvash polycythemia. Haematologica. (2006) 91:744–9. Available online at: http://www.haematologica.org/content/91/6/744.full.pdf+html 16769575

[89] Bond J Gale DP Connor T Adams S de Boer J Gascoyne DM. Dysregulation of the HIF pathway due to VHL mutation causing severe erythrocytosis and pulmonary arterial hypertension. Blood. (2011) 117:3699–701. 10.1182/blood-2010-12-327569 21454469

[90] Hickey MM Richardson T Wang T Mosqueira M Arguiri E Yu H. The von Hippel–Lindau Chuvash mutation promotes pulmonary hypertension and fibrosis in mice. J Clin Invest. (2010) 120:827–39. 10.1172/JCI36362 20197624 PMC2827942

[91] Ball MK Waypa GB Mungai PT Nielsen JM Czech L Dudley VJ. Regulation of hypoxia-induced pulmonary hypertension by vascular smooth muscle hypoxia-inducible factor-1alpha. Am J Respir Crit Care Med. (2014) 189:314–24. 10.1164/rccm.201302-0302OC 24251580 PMC3977726

[92] Sheikh AQ Saddouk FZ Ntokou A Mazurek R Greif DM. Cell autonomous and non-cell autonomous regulation of SMC progenitors in pulmonary hypertension. Cell Rep. (2018) 23:1152–65. 10.1016/j.celrep.2018.03.043 29694892 PMC5959296

[93] Shimoda LA Manalo DJ Sham JS Semenza GL Sylvester JT. Partial HIF-1alpha deficiency impairs pulmonary arterial myocyte electrophysiological responses to hypoxia. Am J Physiol Lung Cell Mol Physiol. (2001) 281:L202–8. 10.1152/ajplung.2001.281.1.L202 11404263

[94] Wang J Weigand L Lu W Sylvester JT Semenza GL Shimoda LA. Hypoxia inducible factor 1 mediates hypoxia-induced TRPC expression and elevated intracellular Ca2+ in pulmonary arterial smooth muscle cells. Circ Res. (2006) 98:1528–37. 10.1161/01.RES.0000227551.68124.98 16709899

[95] Martin E Dahan D Cardouat G Gillibert-Duplantier J Marthan R Savineau JP. Involvement of TRPV1 and TRPV4 channels in migration of rat pulmonary arterial smooth muscle cells. Pflugers Arch Eur J Physiol. (2012) 464:261–72. 10.1007/s00424-012-1136-5 22820913

[96] Vander Heiden MG Cantley LC Thompson CB Understanding the Warburg effect: the metabolic requirements of cell proliferation. Science. (2009) 324:1029–33. 10.1126/science.1160809 19460998 PMC2849637

[97] Liberti MV Locasale JW The Warburg effect: how does it benefit cancer cells? Trends Biochem Sci. (2016) 41:211–8. 10.1016/j.tibs.2015.12.001 26778478 PMC4783224

[98] Fijalkowska I Xu W Comhair SAA Janocha AJ Mavrakis LA Krishnamachary B. Hypoxia inducible-factor1α regulates the metabolic shift of pulmonary hypertensive endothelial cells. Am J Pathol. (2010) 176:1130–8. 10.2353/ajpath.2010.090832 20110409 PMC2832136

[99] Plecitá-Hlavatá L Tauber J Li M Zhang H Flockton AR Pullamsetti SS. Constitutive reprogramming of fibroblast mitochondrial metabolism in pulmonary hypertension. Am J Respir Cell Mol Biol. (2016) 55:47–57. 10.1165/rcmb.2015-0142OC 26699943 PMC4942204

[100] Stenmark KR Tuder RM El Kasmi KC. Metabolic reprogramming and inflammation act in concert to control vascular remodeling in hypoxic pulmonary hypertension. J Appl Physiol. (2015) 119:1164–72. 10.1152/japplphysiol.00283.2015 25930027 PMC4816410

[101] Marsboom G Wietholt C Haney CR Toth PT Ryan JJ Morrow E Lung ^18^F-fluorodeoxyglucose positron emission tomography for diagnosis and monitoring of pulmonary arterial hypertension. Am J Respir Crit Care Med. (2012) 185:670–9. 10.1164/rccm.201108-1562OC 22246173 PMC3326289

[102] Zhao L Ashek A Wang L Fang W Dabral S Dubois O Heterogeneity in lung 18FDG uptake in PAH: potential of dynamic 18FDG-PET with kinetic analysis as a bridging biomarker for pulmonary remodeling targeted treatments. Circulation. (2013) 128:1214–24. 10.1161/CIRCULATIONAHA.113.004136 23900048

[103] Li M Riddle SR Frid MG El Kasmi KC McKinsey TA Sokol RJ. Emergence of fibroblasts with a proinflammatory epigenetically altered phenotype in severe hypoxic pulmonary hypertension. J Immunol. (2011) 187:2711–22. 10.4049/jimmunol.1100479 21813768 PMC3159707

[104] Zhao L Chen C-N Hajji N Oliver E Cotroneo E Wharton J. Histone deacetylation inhibition in pulmonary hypertension: therapeutic potential of valproic acid and suberoylanilide hydroxamic acid. Circulation. (2012) 126:455–67. 10.1161/CIRCULATIONAHA.112.103176 22711276 PMC3799888

[105] Dai Z Li M Wharton J Zhu MM Zhao YY. Prolyl-4 Hydroxylase 2 (PHD2) deficiency in endothelial cells and hematopoietic cells induces obliterative vascular remodeling and severe pulmonary arterial hypertension in mice and humans through hypoxia-inducible factor-2α. Circulation. (2016) 133:2447–58. 10.1161/CIRCULATIONAHA.116.021494 27143681 PMC4907810

[106] Kapitsinou PP Rajendran G Astleford L Michael M Schonfeld MP Fields T. The endothelial prolyl-4-hydroxylase domain 2/hypoxia-inducible factor 2 axis regulates pulmonary artery pressure in mice. Mol Cell Biol. (2016) 36:1584–94. 10.1128/MCB.01055-15 26976644 PMC4859687

[107] Cowburn AS Crosby A Macias D Branco C Colaço RDDR Southwood M. HIF2α-arginase axis is essential for the development of pulmonary hypertension. Proc Natl Acad Sci USA. (2016) 113:8801–6. 10.1073/pnas.1602978113 27432976 PMC4978263

[108] Good RB Gilbane AJ Trinder SL Denton CP Coghlan G Abraham DJ. Endothelial to mesenchymal transition contributes to endothelial dysfunction in pulmonary arterial hypertension. Am J Pathol. (2015) 185:1850–8. 10.1016/j.ajpath.2015.03.019 25956031

[109] Tang H Babicheva A McDermott KM Gu Y Ayon RJ Song S Endothelial HIF-2α contributes to severe pulmonary hypertension by inducing endothelial-to-mesenchymal transition. Am J Physiol Cell Mol Physiol. (2017) 314:ajplung00096.2017. 10.1152/ajplung.00096.2017 PMC5866501 29074488

[110] Kim Y-M Barnes EA Alvira CM Ying L Reddy S Cornfield DN Hypoxia-inducible factor-1 in pulmonary artery smooth muscle cells lowers vascular tone by decreasing myosin light chain phosphorylation. Circ Res. (2013) 112:1230–3. 10.1161/CIRCRESAHA.112.300646 23513056 PMC4005857

[111] Iyer NV Kotch LE Agani F Leung SW Laughner E Wenger RH Cellular and developmental control of O2 homeostasis by hypoxia- inducible factor 1α. Genes Dev. (1998) 12:149–62. 10.1101/gad.12.2.149 9436976 PMC316445

[112] Wigerup C Påhlman S Bexell D. Therapeutic targeting of hypoxia and hypoxia-inducible factors in cancer. Pharmacol Ther. (2016) 164:152–69. 10.1016/j.pharmthera.2016.04.009 27139518

[113] Yu T Tang B Sun X. Development of inhibitors targeting hypoxia-inducible factor 1 and 2 for cancer therapy. Yonsei Med J. (2017) 58:489–96. 10.3349/ymj.2017.58.3.489 28332352 PMC5368132

[114] Courtney KD Infante JR Lam ET Figlin RA Rini BI Brugarolas J. Phase I dose-escalation trial of PT2385, a first-in-class hypoxia-inducible factor-2α antagonist in patients with previously treated advanced clear cell renal cell carcinoma. J Clin Oncol. (2017) 36:JCO.2017.74.2627. 10.1200/JCO.2017.74.2627 29257710 PMC5946714

[115] Dai Z Zhu MM Peng Y Machireddy N Evans CE Machado R Therapeutic targeting of vascular remodeling and right heart failure in PAH with HIF-2α inhibitor. Am J Respir Crit Care Med. (2018) 198:1423–34. 10.1164/rccm.201710-2079OC 29924941 PMC6290950

[116] Zhao L Oliver E Maratou K Atanur SS Dubois OD Cotroneo E. The zinc transporter ZIP12 regulates the pulmonary vascular response to chronic hypoxia. Nature. (2015) 524:356–60. 10.1038/nature14620 26258299 PMC6091855

[117] Gupta N Wish JB. Hypoxia-inducible factor prolyl hydroxylase inhibitors: a potential new treatment for anemia in patients with CKD. Am J Kidney Dis. (2017) 69:815–26. 10.1053/j.ajkd.2016.12.011 28242135

